# Supplementary material for: A tumor-secreted protein utilizes glucagon release to cause host wasting
Source: Cell Discov. 2025 Feb 10;11:11. doi: 10.1038/s41421-024-00762-0 (PMC11808122; doi:10.1038/s41421-024-00762-0)
Supplement: Supplementary file 1 — Supplementary information [file 41421_2024_762_MOESM1_ESM.pdf]

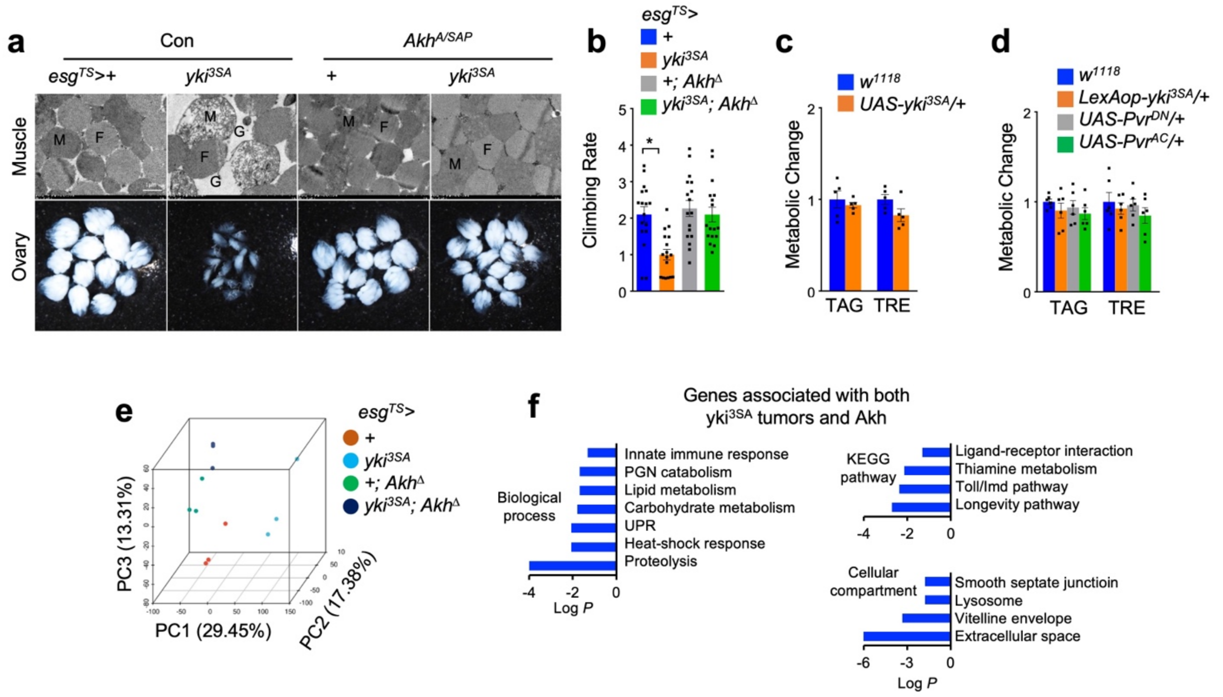

Supplementary Figure 1. Akh is essential for tumor-induced host wasting in *Drosophila*. (**a-b**) Representative images of muscle degeneration (**a,** up) indicated by swollen mitochondria (M) and gaps (G) between mitochondria and myofibril (F), ovary atrophy (**a,** down), and climbing rates (**b,** n=17) of adult yki^{3SA}-tumor-bearing flies with or without *Akh*$^{\Delta}$ mutation (*Akh*^{SAP}/*Akh*^{A}) at day 8. (**c-d**) Metabolic changes including TAG and trehalose (TRE) storages in 8-day old indicated control adult flies (**c,** n=5; **d,** n=6). (**e**) PCA analysis of whole-body gene expression of yki^{3SA}-tumor-bearing flies with or without *Akh*$^{\Delta}$ mutation (*Akh*^{SAP}/*Akh*^{A}) at day 8. (**f**) Gene Ontology Enrichment analysis of 395 differentially expressed genes that are both yki^{3SA}-tumor- and Akh-dependent indicating that the following terms of biological process, cellular compartment, and KEGG pathways are significantly enriched. Data are presented as mean ± SEM. Each dot represents one biological replicate. Statistical analysis was conducted by two-tailed unpaired t-test (**c**) or one-way ANOVA with Bonferroni's multiple-comparisons test (**b, d**). *$p < 0.05$.

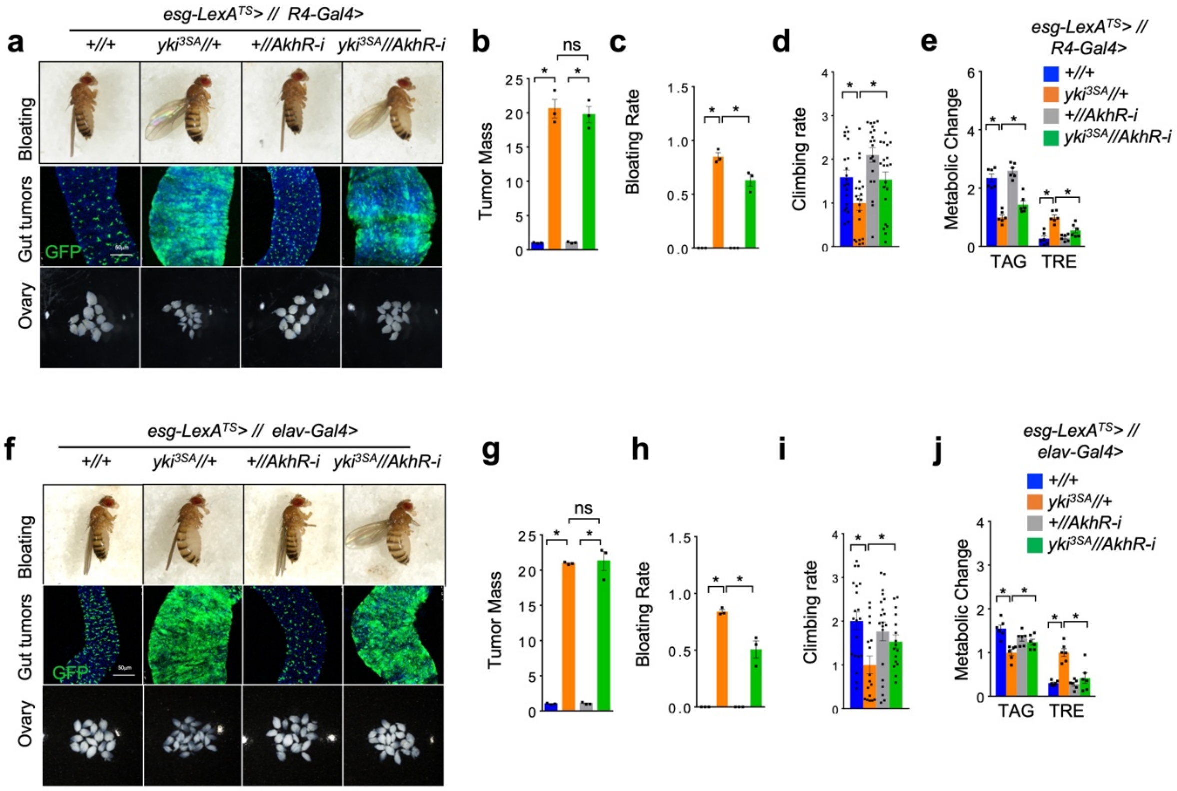

Supplementary Figure 2. **AkhR in either fat body or brain contributes to tumor-induced host wasting.** Representative images of abdomen bloating (**a, f,** up), gut tumors and mass quantification (**a, f,** middle, GFP; **b, g,** n=3), ovary atrophy (**a, f,** bottom), bloating rates (**c, h,** n=3), climbing rates (**d, i,** n=20), and global storages of TAG and trehalose (TRE) (**e, j,** n=6) of adult yki^{3SA} tumor-bearing flies (LexA+*GAL4*) with *AkhR* RNAi in either fat body (*R4-GAL4>*) (**a-e**) or pan-neuron (*elav-GAL4>*) (**f-j**) at day 4. Data are presented as mean ± SEM. Each dot represents one biological replicate. Statistical analysis was conducted by one-way ANOVA with Bonferroni's multiple-comparisons test. *$p < 0.05$.

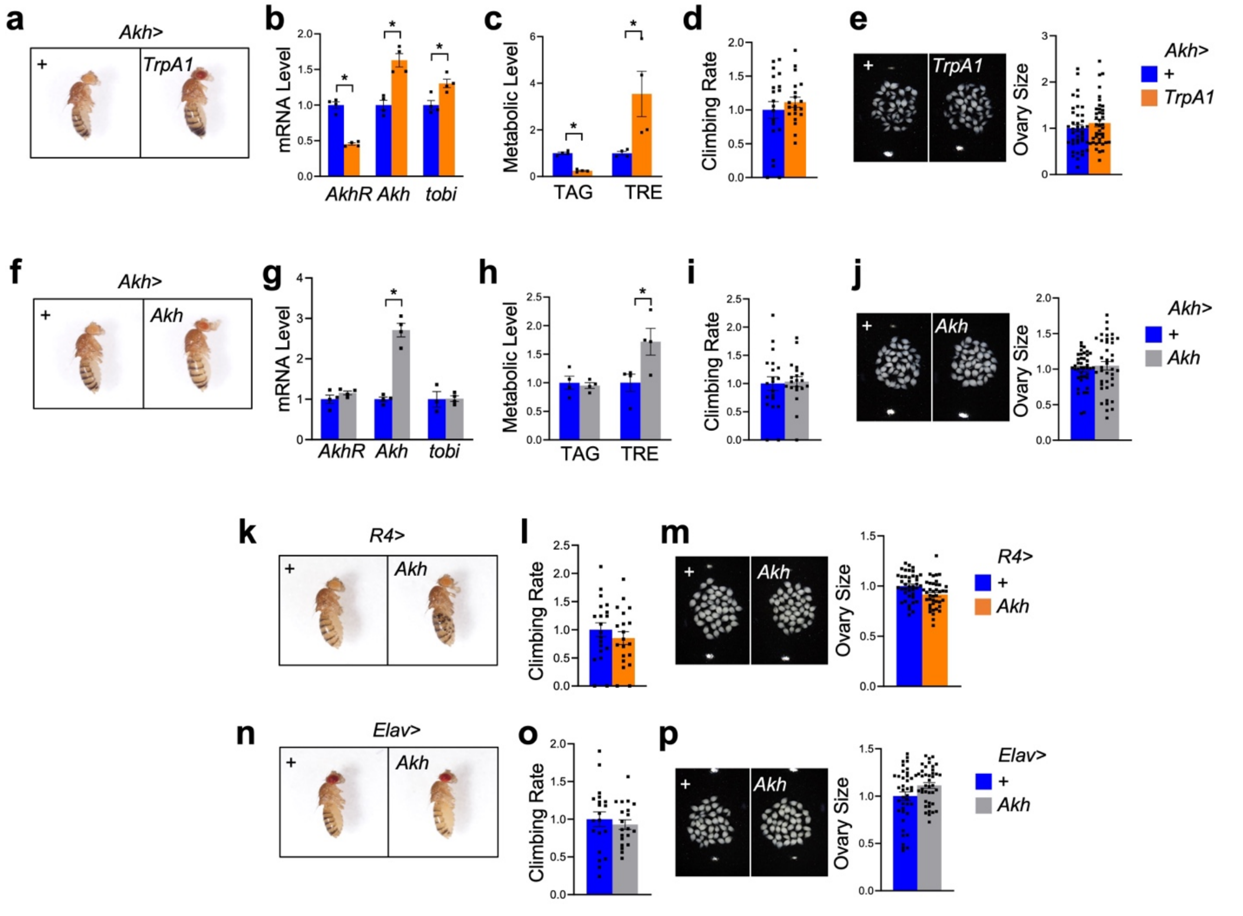

Supplementary Figure 3. Excessive Akh release causes lipid and carbohydrate mobilization but not organ wasting. Representative images of abdomen (**a, f, k, n**), whole-body gene expression (**b, g,** n=4, 5 flies/replicate), whole-body TAG and TRE levels (**c, h,** n=4, 5 flies/replicate), climbing rates (**d, i, l, o,** n=20), as well as ovary images and size quantification (**e, j, m, p,** n>10), of adult flies with TrpA1 overexpression in the APCs (**a-e**), Akh overexpression in the APCs (*Akh-GAL4>,* **f-j**), fat body (*R4-GAL4,* **k-m**), or pan-neurons (*elav-GAL4,* **n-p**) at 29 degree at day 8 (**a-j**) or 25 degree at day 4 (**k-p**). Data are presented as mean ± SEM. Each dot represents one biological replicate. Statistical analysis was conducted by two-tailed unpaired t-test. *$p < 0.05$.

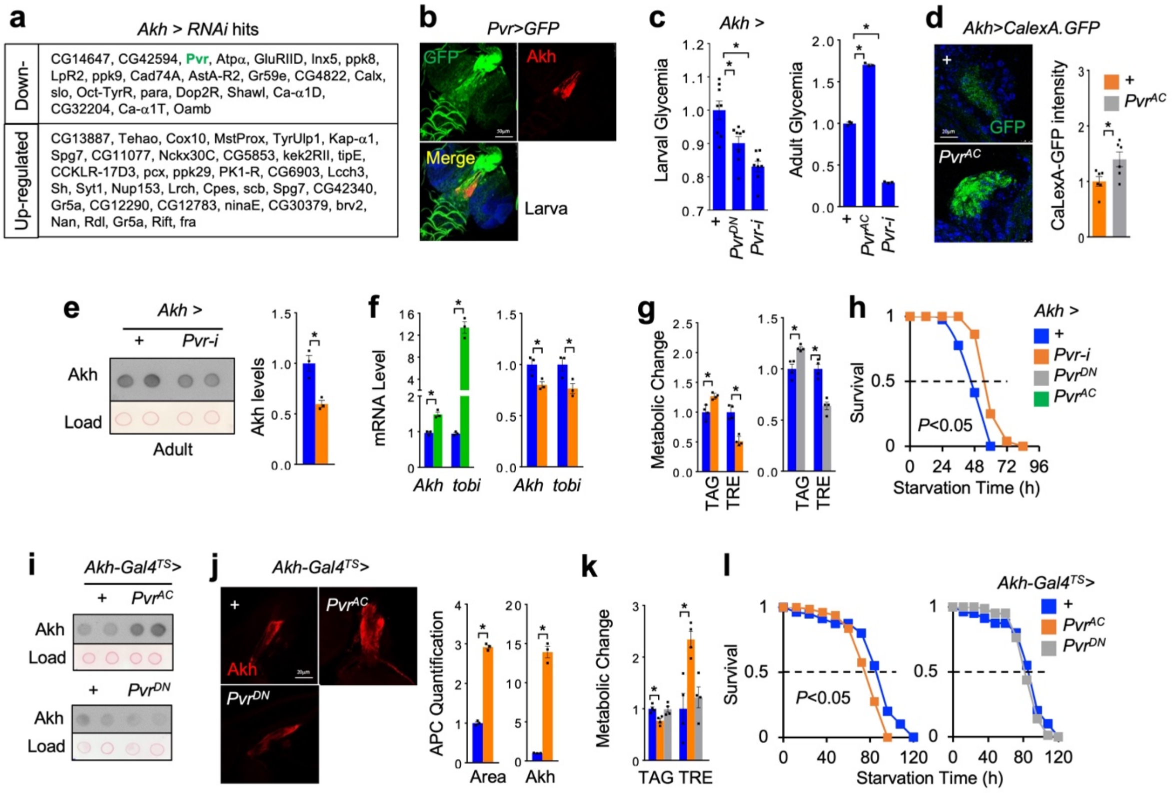

Supplementary Figure 4. Pvr autonomously regulates Akh production. (**a**) RNAi against indicated genes that affect larval glycemic levels by >10% in APCs. (**b**) Immunostaining indicating Pvr expression in the larval APCs (green, Pvr>GFP; red, anti-Akh). (**c**) Glycemic (circulating trehalose) levels of indicated flies (left, larvae at day 5, n=8, 10 flies/replicate; right, adult flies at day 4, n=3, 30 flies/replicate). (**d**) Representative images of CalexA.GFP indicating intracellular Ca^{2+} flux in adult flies with Pvr^{AC} overexpression in the APCs (left) and GFP quantification (right, n=6). (**e-h**) Circulating Akh levels (**e,** left, dot-blot; right, quantification, n=3), systemic mRNA levels of *Akh* and *tobi* (**f**, n=3, 5 flies/replicates), TAG and trehalose (TRE) storages (**g**, n=4, 5 flies/replicates), and survival under starvation (**h,** n=4, 20 flies/replicate) of indicated flies at day 4. (**i-l**) Circulating Akh levels (**i,** dot-blot), Akh production in APCs (**j**, left) and quantification of APC masses and intracellular Akh amounts (**j,** right, n=3), TAG and trehalose (TRE) (**k**, n=4, 5 flies/replicates), and survival under starvation (**l,** n=4, 20 flies/replicate) of indicated flies with Pvr manipulation only in adult APCs using *tub-Gal80^TS^* at day 5 after transgene induction. Data are presented as mean ± SEM. Each dot represents one biological replicate. Statistical analysis was conducted by two-tailed unpaired t-test (**d-g, j-k**) or log-rank test (**h, l**). *$p < 0.05$.

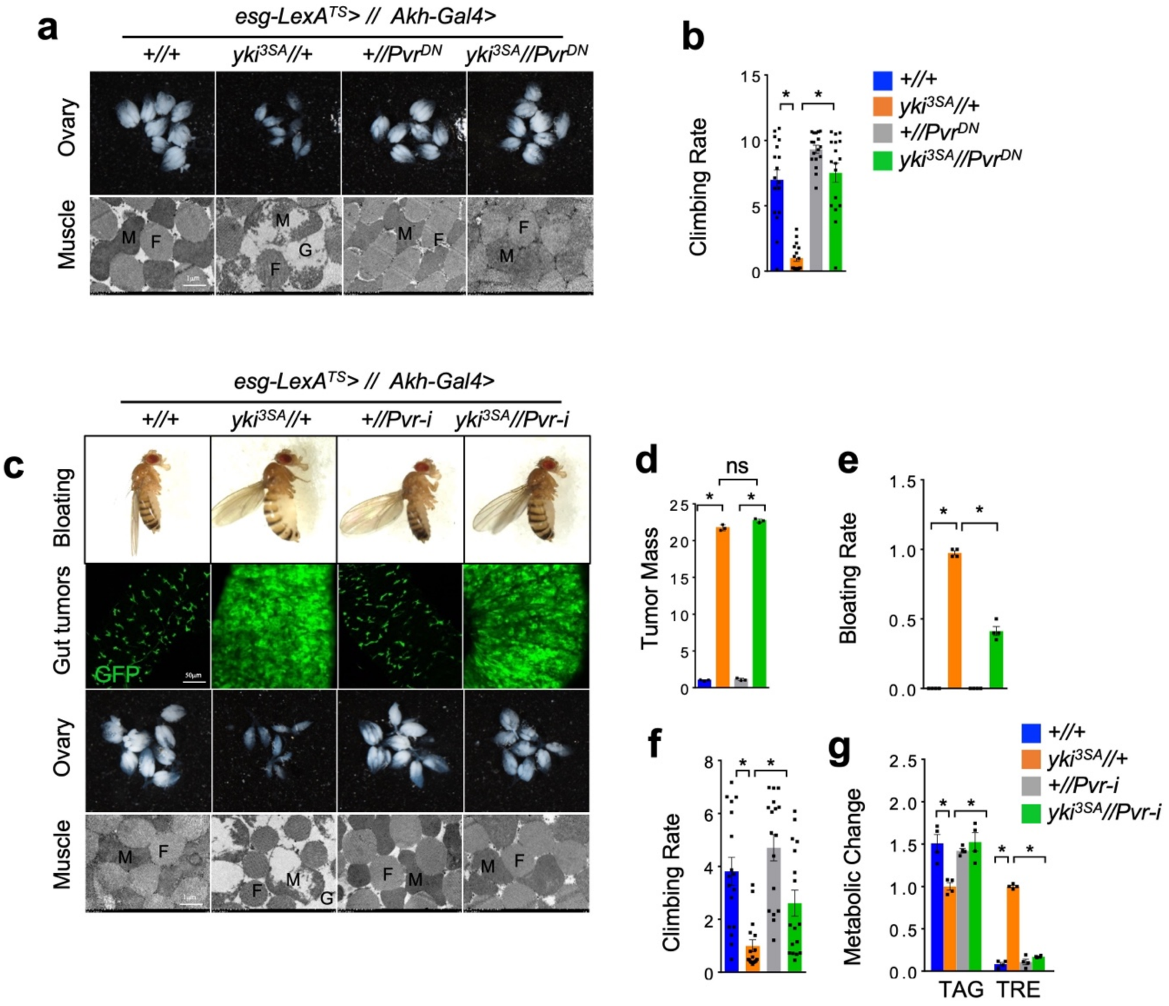

Supplementary Figure 5. Pvr blockade in APCs alleviates tumor-induced wasting. Representative images of abdomen bloating (**c,** up), gut tumors and mass quantification (**c,** middle, GFP; **d,** n=3), ovary atrophy (**a,** up; **c,** middle) and muscle degeneration (**a, c,** bottom) indicated by swollen mitochondria (M) and gaps (G) between mitochondria and myofibril (F), climbing rates (**b,** n=17; **f,** n=17), bloating rates (**e,** n=4), and metabolic changes including TAG and trehalose (TRE) storages (**g,** n=4) of yki^{3SA}-tumor-bearing flies (*LexA+GAL4*) with APC Pvr inactivation (Pvr^{DN}, **a-b**) or Pvr RNAi (**c-g**) at day 6. Data are presented as mean ± SEM. Each dot represents one biological replicate. Statistical analysis was conducted by one-way ANOVA with Bonferroni's multiple-comparisons test. *$p < 0.05$.

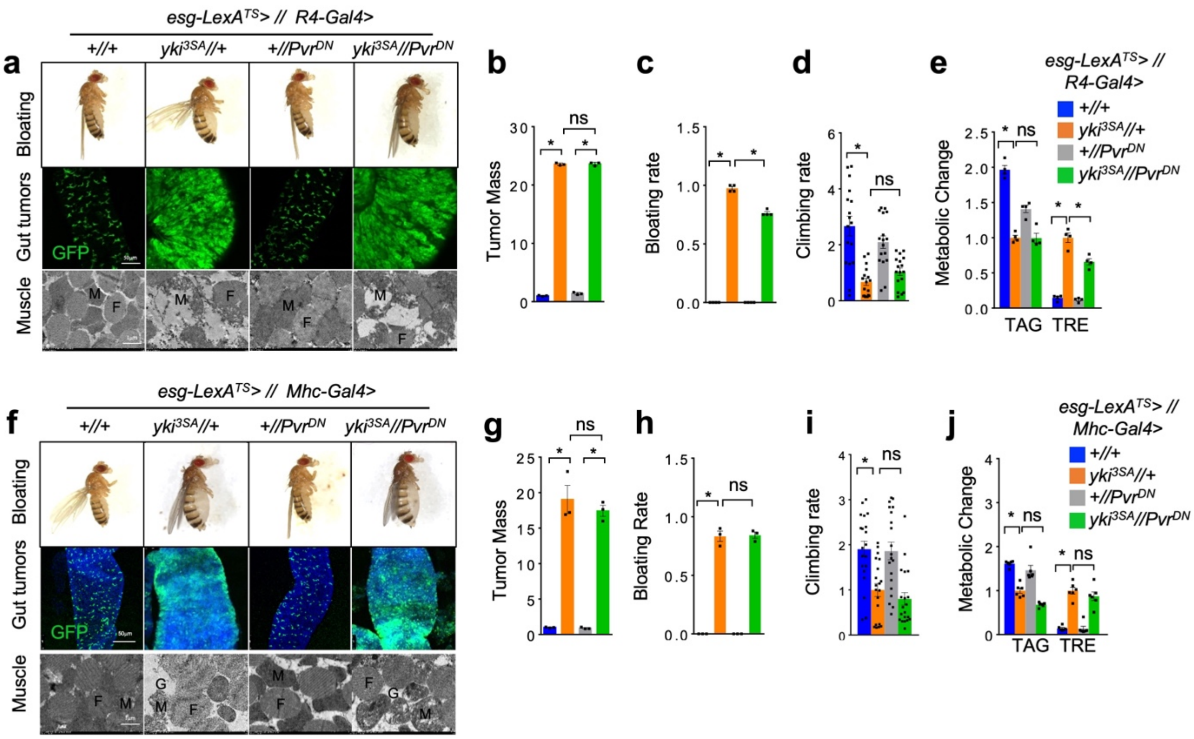

Supplementary Figure 6. Pvr blockade in muscle or fat body hardly alleviates tumor-induced wasting. Representative images of abdomen bloating (**a, f,** up), gut tumors and mass quantification (**a, f,** middle, GFP; **b, g**, n=3), muscle degeneration (**a, f,** bottom) indicated by swollen mitochondria (M) and gaps (G) between mitochondria and myofibril (F), bloating rates (**c,** n=4; **h,** n=3), climbing rates (**d,** n=17; **i,** n=20), and metabolic changes including TAG and trehalose (TRE) storages (**e,** n=4; **j,** n=6) of yki^{3SA}-tumor-bearing flies (*LexA+GAL4*) with Pvr inactivation (Pvr^{DN}) in the fat body (*R4>,* **a-e**) or muscle *(Mhc>,* **f-j**) at day 6. Data are presented as mean ± SEM. Each dot represents one biological replicate. Statistical analysis was conducted by one-way ANOVA with Bonferroni's multiple-comparisons test. *p < 0.05.

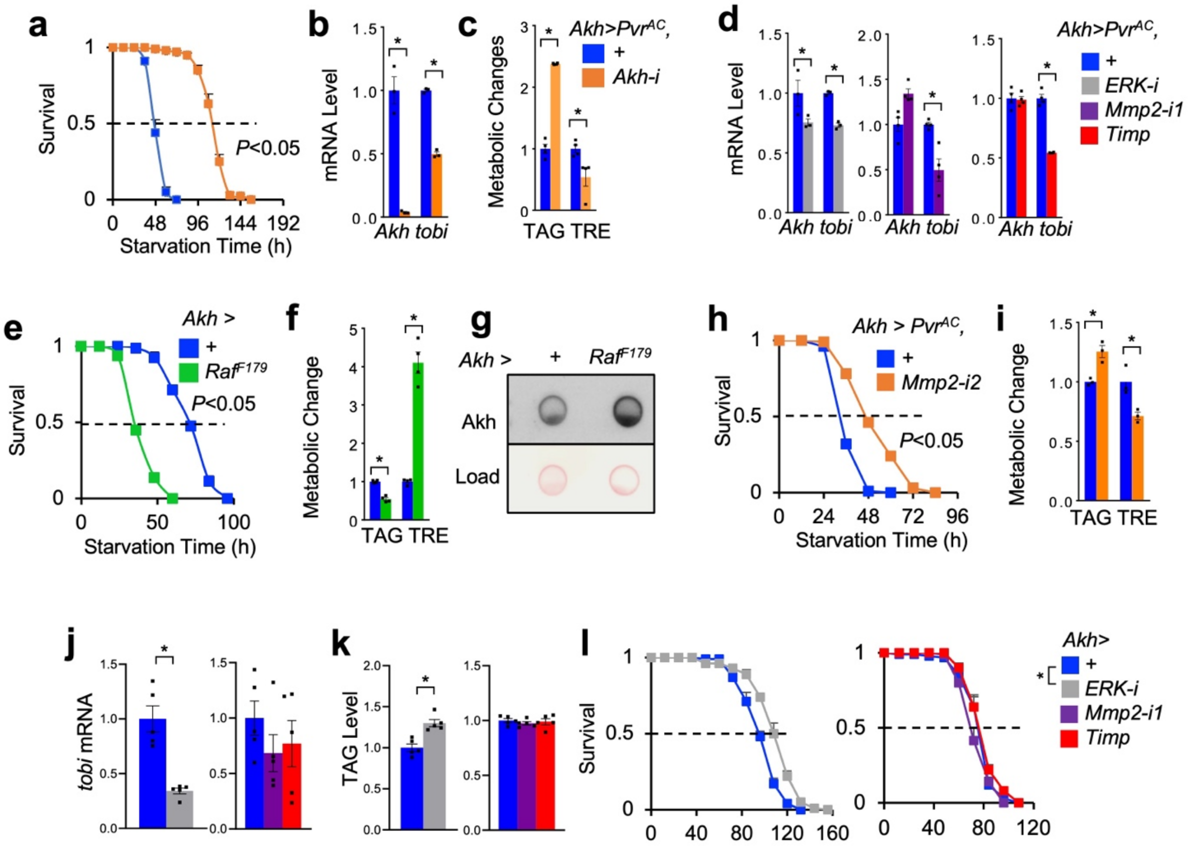

Supplementary Figure 7. Pvr regulation of Akh production. Survival under starvation (**a, e, h, l,** n=4, 20 flies/replicate), whole body *Akh* or *tobi* expression (**b, d, j,** n=3 or 5, 5 flies/replicate), metabolic changes such as TAG or TRE storages (**c, f, i, k,** n=3 or 5, 5 flies/replicate) and circulating Akh levels in the hemolymph (**g**) of adult flies with indicated genotypes at day 4 (**a-i**) or 8 (**j-l**). Data are presented as mean ± SEM. Each dot represents one biological replicate. Statistical analysis was conducted by two-tailed unpaired t-test (**b-d, f, i, j,** left, **k,** left), one-way ANOVA with Bonferroni's multiple-comparisons test (**j,** righ**, k,** right), or log-rank test (**a, e, h, l**). *$p < 0.05$.

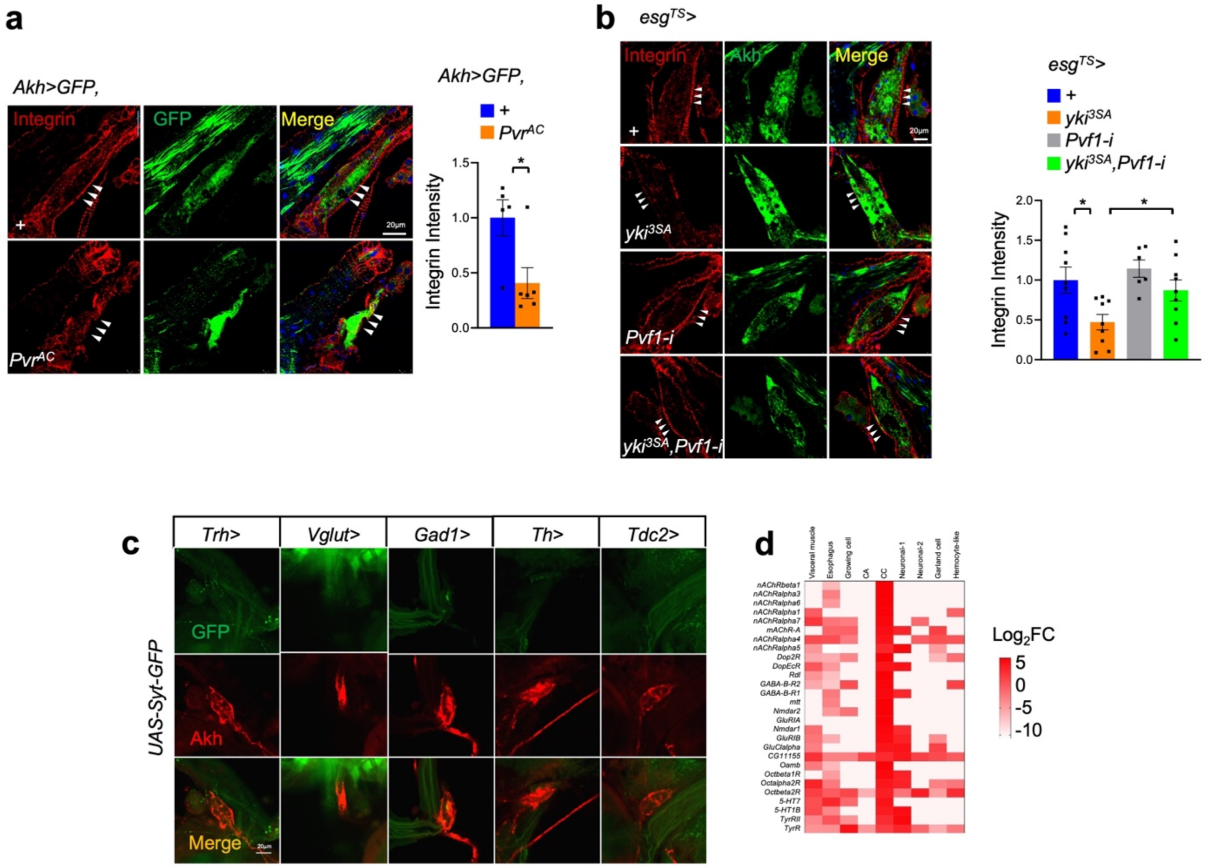

Supplementary Figure 8. ECM homeostasis and neural innervation of APCs. (**a-b**) Representative images of ECM homeostasis indicated by Integrin βPS (**a, b,** left, red, anti-integrin) around the somas of APCs (**a**, left, green, GFP; **b,** left, green, anti-Akh) and quantification of extracellular integrin amounts (**a, b,** n=5-9) of adult flies with Pvr^{AC} overexpression in APCs at day 4 or flies bearing yki^{3SA}-tumor plus *Pvf1 R*NAi at day 7. (**c**) Representative images of boutons that are indicated by Syt-GFP (green) driven by indicated GAL4 lines in the somas of adult APCs (red, Akh) at day 4. (**d**) Published snRNA-seq data indicating that multiple neurotransmitter receptors are expressed in adult CCs or APCs. Data are presented as mean ± SEM. Each dot represents one biological replicate. Statistical analysis was conducted by two-tailed unpaired t-test (**a**) or one-way ANOVA with Bonferroni's multiple-comparisons test (**b**). *p < 0.05.

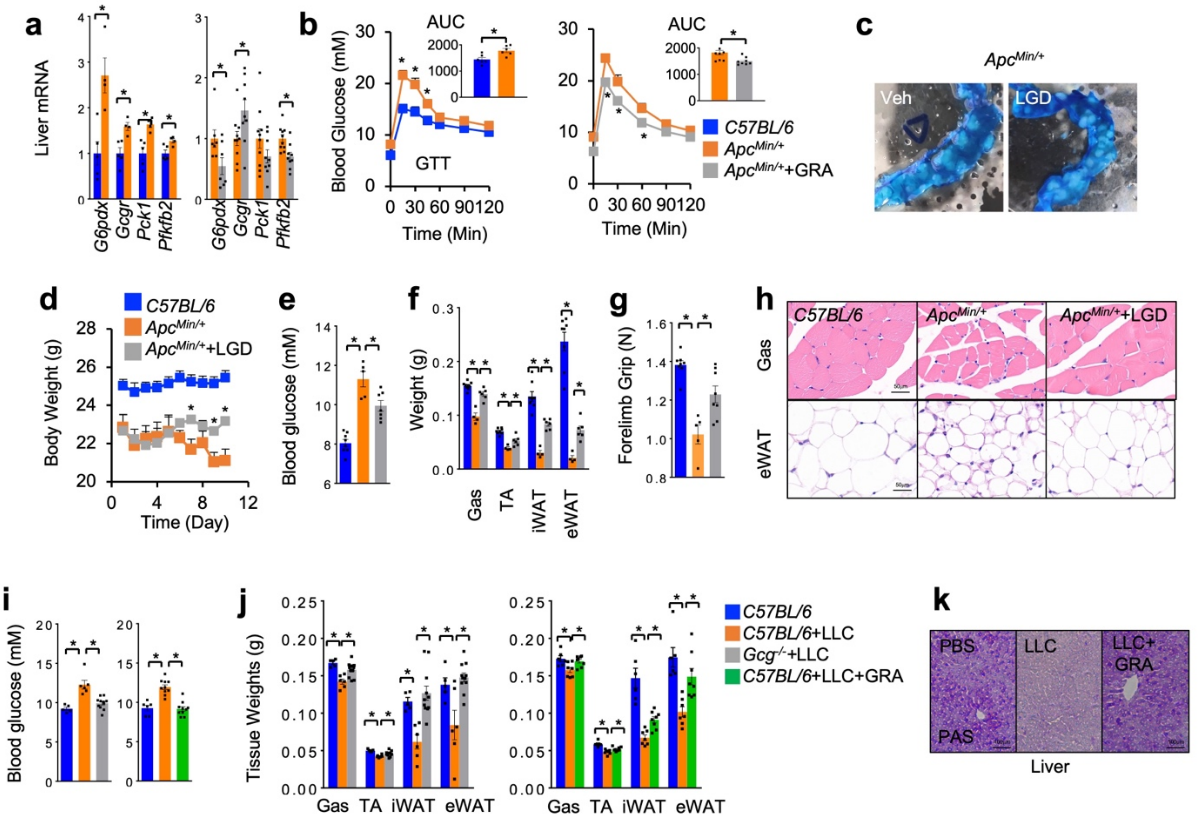

Supplementary Figure 9. **Blockade of glucagon response alleviates wasting in tumor-bearing mice.** (**a-b**) Hepatic gene expression (**a**) and glucose tolerance test (GTT) (**b**) of *Apc*$^{Min/+}$ mice from week 18 with or without daily IP injection of 10 mg/Kg/day GRA Ex-25 (GRA) for two weeks (n=10). (**c-h**) Gut tumors (**c**), body weights (**d**), fed blood glucose (**e**), tissue weights (**f**), forelimb grip strengths (**g**), and tissue morphologies (**h**, Gas, myotube diameters; eWAT, adipocyte sizes) of *Apc*$^{Min/+}$ mice from week 18 with or without IP injection of 6 mg/Kg/day LGD-6972 (LGD) for 10 days (two injections in every three days) for two weeks (n=5). (**i-k**) Fed blood glucose levels (**i**), tissue weights (**j**), and liver glycogen contents (**k**, PAS staining) of indicated LLC-tumor-bearing mice (*C57BL/6*, n=5; *C57BL/6*+LLC, n=6; *Gcg*$^{-/-}$+LLC, n=10) or LLC-tumor-bearing mice with daily IP injection of GRA from day 14 for 7 days (PBS, n=6; LLC, n=7; LLC+GRA, n=8, 10 mg/Kg/day). Data are presented as mean ± SEM. Each dot represents one biological replicate. Statistical analysis was conducted by two-tailed unpaired t-test (**a-b**) or one-way ANOVA with Bonferroni's multiple-comparisons test (**d-g, i-j**). *$p < 0.05$.

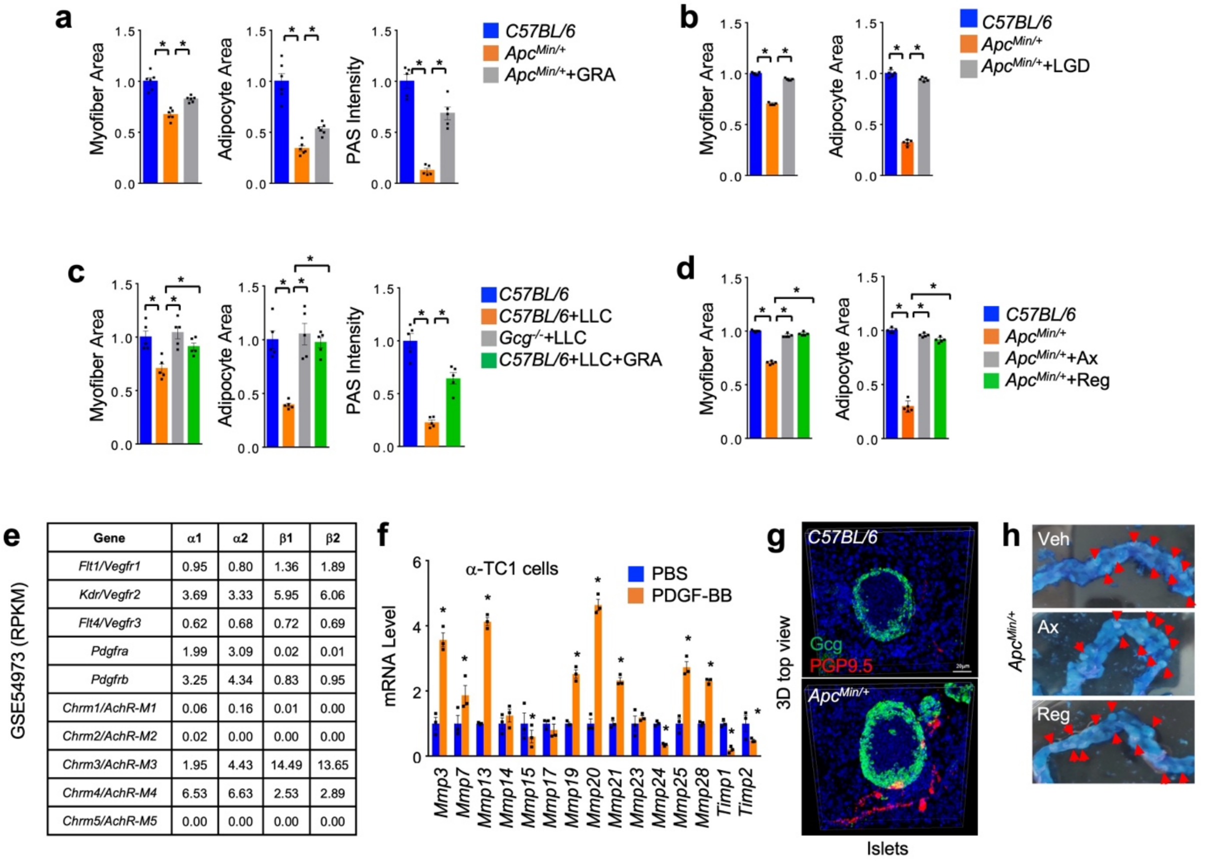

Gene	α1	α2	β1	β2
Flt1/Vegfr1	0.95	0.80	1.36	1.89
Kdr/Vegfr2	3.69	3.33	5.95	6.06
Flt4/Vegfr3	0.62	0.68	0.72	0.69
Pdgfra	1.99	3.09	0.02	0.01
Pdgfrb	3.25	4.34	0.83	0.95
Chrm1/AchR-M1	0.06	0.16	0.01	0.00
Chrm2/AchR-M2	0.02	0.00	0.00	0.00
Chrm3/AchR-M3	1.95	4.43	14.49	13.65
Chrm4/AchR-M4	6.53	6.63	2.53	2.89
Chrm5/AchR-M5	0.00	0.00	0.00	0.00

Supplementary Figure 10. **PDGFR/VEGFR express in α-cells.** (**a-d**) Quantification of gastrocnemius myofiber cross-sectional area, epididymal white adipocyte size, as well as hepatic glycogen content (PAS intensity) in the indicated mice (n=5). (**e**) Gene expression in both mouse α- and β-cells as indicated by RPKM in a published dataset (GSE54973). Genes with RPKM > 1 are considered as functional expressed. (**f**) Gene expression in α-TC1 cells that were treated with 10 ng/mL PDGF-BB for 3 hours (n=3). (**g**) Surface rendering of confocal images merging 8 sections of neve-α-cell contact the islet from the top side (green, Gcg; red, PGP9.5). (**h**) Tumor morphologies indicated by Methylene-blue staining in the colon (arrows indicate tumors) of that were performed daily IP injection of PDGFR/VEGFR inhibitors from week 16 for 2 weeks. *C57BL/6* mice (control), *Apc*^{Min}+Veh, *Apc*^{Min}+Ax (30 mg/Kg/day), and *Apc*^{Min}+Reg (20 mg/Kg/day) (n=5). Data are presented as mean ± SEM. Each dot represents one biological replicate. Statistical analysis was conducted by two-tailed unpaired t-test (**f**) or one-way ANOVA with Bonferroni's multiple-comparisons test (**a-d**). *$p < 0.05$.

Supplementary Table Legends

Supplementary Table 1. The FPKM values of gene expression in the whole body of yki^{3SA}-tumor-bearing flies with or without Akh mutation.

Supplementary Table 2. The glycemic changes of larvae with APCs bearing different RNAi against transmembrane proteins.

Variable	Pancreatic benign diseases (n=15)	Pancreatic cancer without weight decline (n=18)	Pancreatic cancer with weight decline (n=21)	One-Way ANOVA (p Value)
Age (y)	56.3 ± 3.5	57.3 ± 2.9	61/1 ± 2.1	0.4393
Gender (M//F)	7//8	8//10	14//7	0.3110
BMI (Kg/m2)	23.4 ± 0.9	22.4 ± 0.7	19.9 ± 0.6	**0.0073**
Serum Gcg (pg/mL)	343.1 ± 23.6	610.3 ± 83.4	511.4 ± 35.2	**0.0072**
Serum creatinine (μM)	69.5 ± 5.5	59.0 ± 3.6	78.9 ± 13.1	0.319
Serum uric acid (mM)	313.3 ± 36.5	249.2 ± 26.6	286.5 ± 19.6	0.2729
ALT (IU/L)	46.0 ± 18.4	131.2 ± 29.7	183.4 ± 38.8	**0.0181**
AST (IU/L)	52.1 ± 26.7	96.2 ± 19.3	149.4 ± 38.0	0.0935
Blood glucose (mM)	6.0 ± 0.7	6.3 ± 0.6	5.9 ± 0.5	0.8945

>1.5% weight loss per month within three months is defined as weight decline.
Values are means ± standard deviations.
(BMI, Body mass index; Gcg, glucagon; ALT, Alanine aminotransferse; AST, Aspartate aminotransferase)

Supplementary Table 3. Clinical characteristics of patients with pancreatic cancer and benign diseases.

Genes	Primers
Fly *RpL32*	F: GCTAAGCTGTCGCACAAATG
	R: GTTCGATCCGTAACCGATGT
Fly *Akh*	F: TCCCAAGAGCGAAGTCCTCA
	R: CCAGAAAGAGCTGTGCCTGA
Fly *tobi*	F: GTCATGCATCCTGTGTGGTC
	R: GATTTCCAGCTGGCTGTTGT
Mouse *β-actin*	F: AAATCGTGCGTGACATCAAA
	R: AAGGAAGGCTGGAAAAGAGC
Mouse *G6pdx*	F: CCGGTGTTTGAACGTCATCT
	R: CAATGCCTGACAAGACTCCA
Mouse *Pck1*	F: ATCATCTTTGGTGGCCGTAG
	R: ATCTTGCCCTTGTGTTCTGC
Mouse *Pfkfb2*	F: GACAAGCCAACTCACAACTTCC
	R: ACACTGTAATTTCTTGGACGCC
Mouse *Gcgr*	F: TGCACTGCACCCGAAACTAC
	R: CATCGCCAATCTTCTGGCTGT
Mouse *Gcg*	F: CGTGCCCAAGATTTTGTGCA
	R: CCCTTCAGCATGCCTCTCAA
Mouse *Mmp3*	F: GGAAATCAGTTCTGGGCTATACGA
	R: TAGAAATGGCAGCATCGATCTTC
Mouse *Mmp7*	F: GCAGAATACTCACTAATGCCAAACA
	R: CCGAGGTAAGTCTGAAGTATAGGATACA
Mouse *Mmp13*	F: GGGCTCTGAATGGTTATGACATTC
	R: AGCGCTCAGTCTCTTCACCTCTT
Mouse *Mmp14*	F: AGGAGACAGAGGTGATCATCATTG
	R: GTCCCATGGCGTCTGAAGA
Mouse *Mmp15*	F: ATCCCCTATGACCGCATTGAC
	R: CCCCTGCCAGACACTGATG
Mouse *Mmp17*	F: GGCAGTATGTTCCTGCACTTCA
	R: GCTAGCACTGCCCTCAGGAT
Mouse *Mmp19*	F: GCCCATTTCCGGTCAGATG
	R: AGGGATCCTCCAGACCACAAC
Mouse *Mmp20*	F: GATCAGGAGGATTAAGGAGCTACAAA
	R: GGCGGTAGTTAGCCACATCAG

Mouse *Mmp21*	F: TCCAAAGAAGATGAGCCAAGTG
	R: ACGCTGAATCGAGGTTTCTG
Mouse *Mmp23*	F: CAGACTGTTGACCATGTCGGTAA
	R: GAAGGAAAGAACTCTGTATGTGAGGTT
Mouse *Mmp24*	F: TATCATGGCTCCCTTCTACCAATAC
	R: CTGCGGACCGGGAGTGT
Mouse *Mmp25*	F: TGGCTGTCTGGGCTACTGAA
	R: GGTAGGCCCGAGCAAAGTG
Mouse *Mmp28*	F: CCACTTGGACAGAGAGGATCAGT
	R: AAGCGTTTCTTACGCCTCATTT
Mouse *Timp1*	F: CATGGAAAGCCTCTGTGGATATG
	R: AAGCTGCAGGCACTGATGTG
Mouse *Timp2*	F: CCAGAAGAAGAGCCTGAACCA
	R: GTCCATCCAGAGGCACTCATC

Supplementary Table S4. qPCR primers used in this study
